# Proximity of food retailers to schools and rates of overweight ninth grade students: an ecological study in California

**DOI:** 10.1186/1471-2458-11-68

**Published:** 2011-01-31

**Authors:** Philip H Howard, Margaret Fitzpatrick, Brian Fulfrost

**Affiliations:** 1 Department of Community, Agriculture, Recreation and Resource Studies, Michigan State University, 316 Natural Resources, East Lansing, MI, 48824, USA; 2 Design, Community & Environment, 1625 Shattuck Ave, Suite 300, Berkeley, CA, 94709, USA

## Abstract

**Background:**

The prevalence of obesity and overweight in youth has increased dramatically since the 1980s, and some researchers hypothesize that increased consumption of low-nutrient, energy-dense foods is a key contributor. The potential importance of food retailers near schools has received increasing attention, but public health research and policy has focused primarily on fast food restaurants. Less is known about the relationship between overweight/obesity and other types of retailers. This study aims to investigate the potential associations between nearby 1) fast food restaurants, 2) convenience stores, and 3) supermarkets, and rates of overweight students in California schools.

**Methods:**

We examined the rate of overweight ninth grade students in public schools in 2007 using linear regression. The percentage of overweight students per school was determined by a state required physical fitness test, with three different options for measuring individual body composition. Our key independent variables were the presence of three different types of retailers within 800 m network buffers of the schools. Additional independent variables included school ethnic, gender and socioeconomic composition, as well as urban/non-urban location. We obtained the data from the California Department of Education and ESRI, Inc.

**Results:**

The presence of a convenience store within a 10-minute walking distance of a school was associated with a higher rate of overweight students than schools without nearby convenience stores, after controlling for all school-level variables in the regression (1.2%, 95% confidence interval 0.03, 2.36). Nearby fast food restaurants and supermarkets, however, were not associated with school rates of overweight students.

**Conclusions:**

Public health researchers and policy-makers interested in the food environments outside schools should expand their recent focus on nearby fast food restaurants to include convenience stores, which may also be important sources of low-nutrient, energy-dense foods for students.

## Background

The rate of child and adolescent obesity in the United States has increased threefold since 1980, and although currently steady, remains at 17% [1]. Excess weight in children and young adults is associated with greater risks for diabetes, cardiovascular disease and certain cancers [2], as well as associated economic costs[3]. A number of studies have implicated increased consumption of low-nutrient, energy-dense foods such as sugary drinks and fast foods as a factor in elevated rates of overweight and obesity [4-7].

Recently, both researchers and policy-makers have increased their attention on the role of retailers in providing access to these foods as a potential risk factor for increased body fat[8]. Although our understanding of these potential relationships remains limited, several studies focusing on children and young adults in the US have reported associations between food environments and weight status. Residing near fast food restaurants [9] and convenience stores, for example [10,11], is associated with excess weight, while residing near supermarkets is associated with lower weight [11,12]. While not all studies have observed these types of associations [12-15], similar relationships have been reported for the majority of studies involving adults [16-20]. It is hypothesized that the increased availability of low-energy, nutrient-dense foods in supermarkets (e.g. fresh fruits and vegetables) may account for the most typical differences observed among retailer types [18,21].

Other important contexts for children and young adults are the food environments inside and just outside schools, where students may consume up to half of their total food intake [22]. There have been relatively few studies of food environments outside of schools with respect to the outcomes of overweight or obesity, and, as with studies involving adults, more have focused on fast food restaurants than other types of retailers [23]. A statewide study of middle and high school students in California reported a 6% increase in the odds of overweight when attending schools within 800 m of fast food restaurants, as well as increased soda consumption [24]. An ecological study, also conducted in California, reported higher school rates of overweight when fast food restaurants were located within a smaller, .1 mile buffer, after controlling for school-level variables [25]. One of the few studies of multiple types of retailers near schools in the United States found no associations between proximity to fast food restaurants or convenience stores and adolescent overweight in Minnesota, but these variables were associated with increased consumption of sugar-sweetened beverages [26].

The plausibility of convenience stores near schools influencing dietary patterns in children and young adults is strengthened by a recent observational study in Philadelphia: the researchers reported that fourth, fifth and sixth grade students frequently shopped at corner stores before and after school, and were most likely to buy inexpensive sugar-sweetened drinks, chips and candy (averaging approximately 350 kcal per purchase) [27]. We therefore examined associations between the proximity of fast food restaurants, convenience stores and supermarkets to schools in California, and school overweight rates. We hypothesized that 1) proximity to fast food restaurants would be associated with higher school overweight rates, 2) proximity to convenience stores would be associated with higher school overweight rates, and 3) proximity to supermarkets would be associated with lower school overweight rates.

## Methods

### Variables and Data Sources

The dependent variable for this study was the rate of overweight ninth grade students for public schools in California in 2007. The data were obtained from California Department of Education, which administers a physical fitness test (FITNESSGRAM^®^) between February and May of each year. The test is required by state law for public school students in 5^th^, 7^th ^and ninth grade, and in 2007 approximately 90% of schools participated[28]. The test reports students' body composition, as measured by skinfold (preferred method), body mass index (weight in kilograms divided by height in meters squared), or bioelectric impedance analyzers. These three different measurement options are provided to ensure broad participation, and while all are subject to error (typically 3 to 6%), comparisons of different methods report high test-retest reliability [29,30]. The definition of overweight differs slightly from others, such as the Centers for Disease Control. Instead, classification in this category is determined by criterion-referenced gender-, age- and test-specific cut-offs (e.g. skinfold <32 for a 15 year old female; body mass index <24.5 for a 14 year old male). These standards were established by a national advisory panel, convened by the Cooper Institute of Dallas, Texas[29].

Our analysis is at the school-level because the physical fitness test results are publicly available only at this level of aggregation--individual results are confidential. The original dataset included all public schools in California that reported physical fitness test results. To obtain more stable rates of overweight students, we excluded schools with less than 100 students from the analysis. We focused on ninth graders because they are expected to have greater mobility, and greater financial ability to purchase food from nearby retailers, when compared to 7^th ^and 5^th ^graders.

The physical fitness dataset also provided information on the gender and ethnic composition of students tested in each school. We used this information to construct a variable for gender composition, with percentage of male students as the reference category. We also constructed variables for student composition by major ethnic groups, with percentage non-Hispanic white as the reference category, and additional variables for the percentage of students that were Hispanic/Latino, African-American, Asian, Native American (including Alaskan native), Pacific Islander, and those who declined to state their ethnicity.

The California Department of Education was the source for several additional datasets, all of which are also publicly available. One contained the street addresses of schools in the study, which we used in constructing the retailer proximity variables described below. We obtained two other datasets to construct independent variables for 1) percentage of all students (not just ninth graders) in each school receiving free & reduced price meals in 2007, and 2) urban or non-urban school location. The subsidized meal variable was used as a proxy for school-level socioeconomic status, which at the individual-level is known to be strongly associated with overweight. We defined urban schools as those located in large or mid-size cities (based on U.S. Census Bureau classifications) and compared them to schools that were not in these areas, defined as non-urban.

Another publicly available dataset was purchased from Environmental Systems Research Institute, Inc. (ESRI), It was used to construct variables for the presence/absence of three classes of retailers near schools: 1) fast food restaurants, 2) convenience stores, and 3) supermarkets. ESRI provided geocoded information for data that was first collected by the marketing firm InfoUSA, in 2007. We then selected retailers based initially on the following 8-digit National American Industry Classification System (NAICS) codes: Limited Service Restaurants (72221105), Convenience Stores (44512001), Supermarkets and Grocery Stores (44511001). As reported in another study however [31], the NAICS codes were inconsistently applied. For instance, there were many recognizable convenience stores identified with the NAICS code for Grocer Retail (44511003). To reduce misclassification, retail locations in this category that contained the terms 'Quick-,' 'Mini-,' and 'Liquor-' in the company name were recoded into the "convenience stores" category (n = 668). Supermarkets were defined as retailers in the Supermarket and Grocery Stores category with $2 million or more in annual sales, based on the Food Marketing Institute's definition [32]. Because Limited Service Restaurants (72221105) was also an overly broad category, those with five or more locations were selected as "fast food restaurants," or business chains that provide low price meals without table service. This category included all of the major fast food chains (e.g. McDonald's, Burger King, Taco Bell, Domino's). For the entire state of California we identified 3,646 supermarkets, 4,069 convenience stores, and 20,668 fast food restaurants.

### GIS analysis

The point locations of the schools were geocoded with the Streetmap USA (2006) dataset provided by ESRI, based on street addresses. We validated the accuracy of geocoded locations by spot-checking 10% of the schools. We found the addresses of these schools through web searches and re-geocoded them using Mapquest, an online geocoding service owned by America Online, Inc. [33]. Our spot-checking indicated more than 99% accuracy in our geocoded school locations.

Most previous studies of food proximity have not incorporated the actual pedestrian walking network into their analyses of proximity or food access. Instead they rely on Euclidean (straight-line, circular radius) buffers that do not account for the street network, sidewalks, and other elements of the areas surrounding schools where walking is most feasible [24,31,34,35]. The result is that the area covered by Euclidean buffers can be substantially more than the area covered by equivalent distance network buffers. This can lead to erroneous or misleading results. To improve accuracy in this study we utilized network buffers along the actual street network for the entire state of California.

We used ESRI's Network Analyst extension to ArcView 9.1 to create 800 m network buffers around the final geocoded school points. This distance was selected because it is approximately a ten-minute walk, and is commonly used in other studies of retail food access near schools [24,31,35,36]. These network buffers included both the street lines making up the 800 m street networks around each school, as well as polygons that contained the area encompassing these networked buffers. Once the network buffers were created, we performed a spatial join to calculate the number of fast food, convenience and supermarket retailers located within each of these network buffers. The final result of our GIS analysis was a matrix denoting the presence or absence of each of three classes of retailers within 800 m along the street network for each school. These data were exported out of the GIS for statistical analysis.

### Statistical analysis

We analyzed all data with SPSS (version 15.0.1). Our analysis included descriptive statistics, correlations (Kendall's tau-b), and linear regression. Our first model regressed the dependent variable of school rate of overweight ninth grade students on the three types of nearby retailers assessed in the GIS analysis. Our second model included the additional school-level variables (ethnic, socioeconomic and gender composition, and urban/non-urban location) for comparison.

Prior to regression, we applied logarithm transformations to school ethnic composition variables, with the exceptions of the two largest groups (Hispanic/Latino and White, non-Hispanic), in order to meet assumptions of normality. We also employed multiple imputation as a strategy for dealing with missing data for two independent variables: percentage of students receiving free & reduced price meals, and urban/non-urban school location. This involved utilizing information from the existing data to generate plausible values for missing data, while also representing uncertainty by generating five different data sets with imputed values. These values were imputed with the software Amelia II (version 1.2), and combined for analysis using the procedures detailed by King *et al*.[37]

## Results

### Descriptive statistics

Table 1 shows the descriptive characteristics of the study. The sample consisted of 879 public schools in California with scores for 100 or more ninth grade students on the California Physical Fitness Test in 2007 (659 schools reporting scores for fewer ninth grade students were excluded from the analysis; 479 of these excluded schools reported overweight scores, with a mean rate of 33.8%). The number of students per school ranged from 100 to 1486. The percentage of students that were overweight in each school ranged from 3.0 to 75.4%, with a mean of 30.7%. Although this is a substantial range, it is not surprising given the heterogeneity in ethnic and income and composition of these schools (e.g. up to 91.4% white, non-Hispanic, or 100% Hispanic/Latino). Half of the study schools were located within an 800 m network buffer of a fast food restaurant. The other two types of retailers examined were less prevalent, with 28.6% of schools located near convenience stores, and 23.8% near supermarkets.

**Table 1:** Descriptive statistics for school variables in the analysis (n = 879)

	Percentage	Mean (SD)	Min	Max	Percentage Missing Prior to Imputation
**Dichotomous variables**					
Retailers^a^					0
Fast Food	50.4				
Convenience	28.6				
Supermarket	23.8				
School location					8.8
Non-urban	52.6				
Urban	38.7				
**Continuous variables**					
Number of students per school		474.2 (229.6)	100	1486	0
% Overweight		30.7 (10.4)	3.0	75.4	0
% Male		50.9 (4.5)	26.6	81.0	0
% Female		49.1 (4.5)	19.0	73.4	0
% White, non-Hispanic		32.8 (25.6)	0	91.4	0
% Hispanic/Latino		45.2 (27.3)	1.6	100.0	0
% African-American		7.3 (9.4)	0	79.0	0
% Asian		12.0 (14.8)	0	86.8	0
% Native American		0.84 (1.4)	0	14.9	0
% Pacific Islander		0.7 (1.0)	0	8.6	0
% Declined to state ethnicity		1.5 (3.0)	0	45.7	0
% Free/reduced meals		42.1 (26.0)	0	100.0	6.4

^a ^At least one such retailer within an 800 m network buffer.

### Correlations

Rates of overweight ninth grade students were positively associated with the presence of nearby convenience stores and fast food restaurants, as shown in Table 2. Convenience stores demonstrated stronger correlations with school overweight rates (.138) than fast food restaurants (.091). Supermarkets demonstrated no association with school overweight rates, however.

**Table 2:** Correlations (Kendall's tau-b) for key school variables in the analysis (n = 879).

	Overweight (school %)	**Fast Food**^**a**^	**Convenience**^**a**^	**Supermarket**^**a**^
Fast Food^a^	.091**			
Convenience^a^	.138**	.337**		
Supermarket^a^	-.001	.426**	.231	
Number of Students	-.013	.038	-.028	-.016
% Female	.020	-.019	.007	-.019
% Hispanic/Latino	.494**	.101**	.137**	.020
% African-American (log)	.161**	.072**	.008	-.032
% Asian (log)	-.233**	.016	-.082**	.002
% Native American (log)	-.112**	-.100**	-.060*	-.051
% Pacific Islander (log)	-.055*	.039	-.065	-.043
% Declined to state (log)	-.162**	-.043	-.059*	.030
% Free/reduced meals	.494**	.108**	.131**	.003
Non-urban location	-.083**	-.137**	-.057	-.058

*p < .05, **p < .01

^a ^At least one such retailer within an 800 m network buffer.

Schools with higher percentages of Latino students were more likely to be near convenience stores (.137) and fast food restaurants (.101), but were not more likely to be near supermarkets (.020). Schools with more low-income students, as measured by the percentage receiving free & reduced price meals, demonstrated very similar correlations (.131 with convenience stores, .108 with fast food, .003 with supermarkets). Schools located in non-urban areas were less likely to be near fast food restaurants (-.137) and had lower rates of overweight students (-.083) when compared to urban schools.

### Regression

The first model regresses the percentage of overweight ninth grade students on the three retail variables, as shown in Table 3. It indicates that controlling for the influence of these retailers simultaneously, the presence of a convenience store within an 800 m network buffer of a school is predicted to increase the percentage of overweight students by 3.5% (95% confidence interval 1.9, 5.2). For a typical-sized school (474 ninth grade students), for example, approximately 17 additional students in this grade would be expected to fall in the overweight category in comparison to school without a nearby convenience store. The presence of a fast food restaurant within this same distance is predicted to increase the percentage of overweight students by 1.8% (95% confidence interval 0.2, 3.4), approximately half the strength of the convenience store association. The presence of a supermarket within this buffer is predicted to decrease the number of overweight students by 1.9% (95% confidence interval -3.6, -0.1). The model itself explains 3% of the variation in rates of overweight students.

**Table 3:** Regression of school percentage of overweight ninth grade students on nearby retailers

	B	95% CI	β	SE
(Constant)	29.19	(27.20, 30.17)		0.50
Fast Food^a^	1.80	(0.24, 3.35)	0.09	0.79
Convenience^a^	3.54	(1.94, 5.14)	0.15	0.81
Supermarket^a^	-1.86	(-3.62, -0.09)	-0.08	0.90
				
F	10.60			
Adjusted R-squared	0.03			

^a ^At least one such retailer within an 800 m network buffer.

The second regression model is shown in Table 4. This model includes additional school-level variables that are known to be associated with overweight status. The variation explained was 51%, a substantial increase when compared to the first model. After controlling for these additional variables, the associations between retailers and school overweight rates were weaker, and only the association with convenience stores remained. The presence of a convenience store near a school is predicted to increase its overweight rate by 1.2%. This is approximately 1/3 of that reported in model 1, but the 95% confidence interval (0.03, 2.4) continues to indicate a positive relationship. For a school with the mean number of ninth graders (474), the model predicts that an additional 6 students would be overweight in comparison to a school without a nearby convenience store. The associations with fast food restaurants and supermarkets when controlling for additional variables are much smaller, and their 95% confidence intervals include zero.

**Table 4:** Regression of school percentage of overweight ninth grade students on nearby retailers, including additional school-level variables

	B	(95% CI)	β	SE
(Constant)	15.70	(8.88, 22.32)		3.42
Fast Food^a^	-0.04	(-1.18, 1.10)	-0.01	0.58
Convenience^a^	1.20	(0.03, 2.36)	0.05	0.59
Supermarket^a^	-0.33	(-1.61, 0.95)	-0.01	0.65
% Female	-0.03	(-0.14, 0.08)	-0.01	0.05
% Hispanic/Latino	16.17	(12.83, 19.51)	0.43	1.70
% African-American (log)	5.81	(4.45, 7.16)	0.23	0.69
% Asian (log)	-1.84	(-3.22, -0.46)	-0.08	0.70
% Native American (log)	4.61	(1.81, 7.41)	0.09	1.43
% Pacific Islander (log)	0.96	(-2.18, 4.09)	0.02	1.60
% Declined to state ethnicity (log)	1.53	(-0.63, 3.69)	0.04	1.10
% Free/reduced meals	11.08	(7.48, 14.68)	0.28	1.83
Non-urban location	0.34	(-0.79, 1.46)	0.02	0.57
				
F	75.89			
Adjusted R-squared	0.51			

^a ^At least one such retailer within an 800 m network buffer.

Variables that had stronger positive associations with school overweight rates than nearby convenience stores included the percentage of students receiving free or reduced price meals, and percentage of Latino, African-American and Native American students. The percentage of Asian-American students demonstrated a stronger negative association with obesity rates. Variables in the model that were more weakly associated with school overweight rates than nearby convenience stores included gender composition, percent Pacific Islander or "declined to state" ethnicity, and urban/non-urban location.

## Discussion

This study indicated that schools within 800 m of convenience stores had higher rates of overweight students than schools located farther from these retailers. This association was independent of ethnic, socioeconomic and gender composition, as well as urban/non-urban location. It extends similar findings reported in two studies of children that focused on a different context, retailers surrounding homes. One involved 8^th ^and 10^th ^grade students nationally,[11] and the other focused on 6-8 year old children in East Harlem, NY[10], but both reported that residing near convenience stores was associated with a higher body mass index.

Our analysis also underscores the importance of socioeconomic variables on the risks of being overweight. We observed that 1) the percentage of Hispanic/Latino students, 2) the percentage of students receiving free & reduced price meals, and 3) the percentage of African-American students, demonstrated much stronger associations with school overweight rates than the other variables that we tested. Importantly, the first two of these variables were associated with an increased likelihood that a school would be located near a convenience store. While inequalities in retail food environments outside of schools were not a focus of this study, the results provide further support for income and ethnic disparities reported by others [31,34,35,38]. The potential risks of having convenience stores near schools, even if substantially smaller than known individual/household-level risks by comparison, are nonetheless disproportionately borne by those already at much higher risk of being overweight.

While two recent studies found that residing near supermarkets was associated with a lower risk of overweight,[11,12] our study of the environments surrounding schools did not find an association between the proximity of these retailers and rates of student overweight. Also, in contrast to two previous studies in California [24,25], we failed to find a consistent association between school overweight rates and nearby fast food restaurants. Our results therefore provide only partial support for theories that the availability of low-energy, nutrient-dense foods and/or low-nutrient, energy-dense foods contributes to differing consumption patterns and weight outcomes in students. Some of the limitations of our approach, described below, may account for why these expected associations were not observed, however.

### Study limitations

Our study has a number of limitations. Its ecological design limits inferences about school food environments' potential relationships with individual-level behaviors/outcomes. Even at the school-level we lacked data for variables known to influence food/beverage consumption and physical activity, such as the type of food service [39], presence or absence of vending machines [40], open/closed campus [31], mode of transportation (e.g. buses), and physical education requirements [41]. In addition, the cross-sectional design did not allow for analysis of the time order of exposures and the outcome. Another potential limitation was the exclusion of schools with small numbers of students due to unstable rates. Our sources of data are prone to error, such as those we utilized to classify and geographically locate the retailers, as well as the methods and standards used to determine student overweight status. This error may bias the estimates toward or away from the null hypothesis.

### Study strengths

Our study also has several strengths. We utilized network buffers to more accurately model retailer distance to schools when compared to studies that have employed Euclidean buffers. We simultaneously examined associations between three different retail types and the outcome of overweight, rather than focusing on a single type of retailer. Finally, our outcome measure of student overweight, although aggregated at the school-level, was based on criterion-referenced, test-specific measurements, instead of less accurate student self-reported height and weight.

### Suggestions for future research

Future studies could address some of the limitations of our analysis by ground-truthing retail location data, as well as incorporating additional school-level variables that may influence student energy intake and expenditure. Subsequent efforts could also benefit from characterizing the quantity and types of foods available within restaurants and stores, rather than relying on retailer categories, as otherwise similar establishments may differ greatly in their availability of healthy and less healthy foods [42,43]. Additional methodological improvements could include longitudinal designs and/or cross-classified multilevel models [44]. The latter could incorporate the retail food environments of both schools and residences at the macro-level, and examine how these interact with individual student health outcomes and behaviors, as in a recent study conducted by Laska *et al. *with adolescents in Minneapolis/St. Paul [26].

## Conclusions

This ecological study in California found that nearby convenience stores were associated with higher school rates of overweight students, after controlling for socioeconomic and ethnic factors known to be linked to this outcome. Contrary to our expectations, we did not find strong support for an association between nearby fast food restaurants and higher school rates of overweight students, nor for an association between nearby supermarkets and lower rates of overweight students. The limitations of our study suggest that these findings should be interpreted with caution. They do indicate, however, that public health researchers and policy-makers interested in the food environments outside schools should expand their recent focus on nearby fast food restaurants to include convenience stores, as these may also be important sources of low-nutrient, energy-dense foods for students.

## Competing interests

The authors declare that they have no competing interests.

## Authors' contributions

PHH conceived of and designed the study, participated in its coordination and drafted the manuscript. MF conducted data preparation and statistical analysis, and assisted with drafting the manuscript. BF conducted the GIS analysis and assisted with drafting the manuscript. All authors read and approved the final manuscript.

## Pre-publication history

The pre-publication history for this paper can be accessed here:

http://www.biomedcentral.com/1471-2458/11/68/prepub
